# Evaluation of Digit Ratios in Youth With Polycystic Ovary Syndrome

**DOI:** 10.7759/cureus.67168

**Published:** 2024-08-19

**Authors:** Ferda Evin, İlkay Ayrancı

**Affiliations:** 1 Pediatric Endocrinology, Çiğli Training and Research Hospital, Izmir, TUR

**Keywords:** digit ratio, hyperandrogenism, prenatal androgen exposure, polycystic ovary syndrome, anatomical marker

## Abstract

Introduction: Polycystic ovary syndrome (PCOS) is a common syndrome often observed during adolescence, characterized by ovulatory dysfunction and hyperandrogenism. It is determined that, when female fetuses are exposed to high levels of androgens, it increases their likelihood of developing PCOS in later ages. The 2D:4D digit ratio, which measures the length of the index finger compared to the ring finger, is a precise anatomical indicator of the degree of prenatal androgen exposure. Higher digit ratios in individuals have been associated with outcomes typically attributed to females. In the adolescent age group, the relationship between PCOS and androgen exposure during the antenatal period is not clear.

Aim: The study was aimed to evaluate digit ratios in adolescents with PCOS.

Methods: The study included 38 adolescent girls with PCOS, and 40 healthy adolescent girls were selected as the control group. The digit ratio (2D:4D) was evaluated by digital calipers, and the digit ratios of the patient and control groups were compared.

Results: The mean age in the PCOS group was 15.99±1.18 years, while the control group had a mean age of 16.02±1.06 years. The right-hand 2D:4D digit ratio was significantly lower in the PCOS group (0.93±0.02) compared to the control group (1.00±0.01, p<0.001). Similarly, the left-hand 2D:4D digit ratio was also lower in the PCOS group (0.98±0.03) compared to the control group (1.00±0.01, p<0.001). There was a moderate negative correlation between the left-hand 2D:4D ratio and the modified Ferriman-Gallwey score (mFGS) (r=0.53, p=0.01). Nevertheless, there was not a significant association found between the 2D:4D ratio of the right hand and mFGS.

Conclusion: This study demonstrates that PCOS patients have significantly lower both-hand 2D:4D ratios than healthy controls, suggesting prenatal androgen exposure. Recognizing anatomic markers in adolescence may predict the development of PCOS. The findings align with previous research linking low digit ratios to androgen exposure and various reproductive outcomes.

## Introduction

Polycystic ovary syndrome (PCOS) is a prevailing condition observed in adolescence, characterized by ovulatory dysfunction and hyperandrogenism [1]. PCOS is considered a multifactorial syndrome involving both genetic and environmental factors, typically manifesting during puberty when mature gonadotropin levels are achieved [2]. The primary issue in PCOS pathogenesis is insulin resistance coupled with compensatory hyperinsulinemia. Additional contributing factors include genetic predisposition, beta cell dysfunction, abnormal ovarian and adrenal steroidogenesis, changes in steroid metabolome, neuroendocrine and environmental influences, epigenetic modifications, and an impaired response to energy excess or restriction [3,4]. High androgen exposure to female fetuses is known to play a role in developing PCOS later in life. This exposure can affect the production of steroids, the signaling of insulin, the function of pancreatic β-cells, the interaction between the hypothalamus and pituitary gland, the patterns of neuroendocrine secretion, and the modifications of epigenetic factors.

The digit ratio, specifically the ratio of the index finger to the ring finger (2D:4D), serves as a measurable anatomical marker for prenatal androgen exposure. Previous researchers showed that male fetuses with higher testosterone levels tend to have lower digit ratios compared to females [5]. The 2D:4D ratio has been linked to various traits and conditions, such as autism, dementia, sports performance, aggression, academic achievement, personality traits, skills, sexual orientation, and several diseases. Higher digit ratios in individuals have been associated with outcomes typically attributed to females, supporting the notion that 2D:4D is an indicator of prenatal testosterone exposure [6].

In the adolescent age group, the relationship between PCOS and androgen exposure during the antenatal period is not clear. Given the limited research on the relationship between digit ratio and PCOS in adolescents, this study aims to evaluate digit ratios in adolescent girls diagnosed with PCOS.

## Materials and methods

This study included medical records of 38 adolescents with PCOS at Çiğli Training and Research Hospital from December 2021 to December 2023 retrospectively. Digit measurements were made at the last visits. The study obtained approval for ethics from the hospital's Ethical Committee, with an approval date of 15.11.2023 and a decision number of 1310. All procedures adhered to the ethical rules and standards outlined in the Declaration of Helsinki. Patients' families were informed about the study's purposes and the potential publication of their medical data, with informed written consent obtained from parents.

Participants were girls aged 14-18 years, diagnosed with PCOS at least two years post menarche. PCOS diagnosis was based on Fauser et al.'s 2023 PCOS recommendations [7]. It is necessary to have the following conditions: oligo-ovulation/anovulation and clinical or laboratory hyperandrogenism. Exclusion criteria included conditions such as congenital adrenal hyperplasia, androgen-secreting tumors, use of exogenous androgens, and Cushing’s syndrome that could cause hyperandrogenism. Additionally, cases with congenital disorders and skeletal anomalies were excluded. Clinical parameters assessed included menstrual cycle frequency and duration, modified Ferriman-Gallwey score (mFGS), auxological measurements, and their standard deviation scores (SDS). 

For the control group, healthy adolescents with regular menstrual cycles and no history of chronic disorders were matched by age. These controls were selected from routine health visit cases. All auxological data were calculated using an automatic calculator [8].

The lengths of the index and ring fingers on the ventral surface have been determined by digital vernier calipers with a resolution of 0.01 mm to find the digit ratios. All measurements were conducted by the same pediatric endocrinologist.

Hormone levels from blood samples taken on the 3rd-5th day of menstruation, including dehydroepiandrosterone sulfate (DHEAS), follicle-stimulating hormone (FSH), luteinizing hormone (LH), total testosterone, estradiol (E2), prolactin, and thyroid function tests, were analyzed using the chemiluminescence method on the Beckman Coulter DxI® 600 analyzer.

Statistical Product and Service Solutions (SPSS, version 25.0; IBM SPSS Statistics for Windows, Armonk, NY) was employed to conduct the analysis. The Shapiro-Wilk method was used to verify the conformity of numerical variables with the normal distribution. Descriptive statistics are presented as the mean ± standard deviation for variables that are normally distributed and as the median (range) for skewed data. The Mann-Whitney U test was applied for skewed data, while the independent samples t-test was employed for normally distributed variables. Group comparisons were conducted accordingly. Statistical significance was defined as a p-value of less than 0.05. The multiplicity of statistical tests was not adjusted.

## Results

Thirty-eight adolescent girls diagnosed with PCOS were included in the patient group, and 40 healthy adolescent girls constituted the control group. The mean age of the PCOS group was 15.99±1.18, and the mean age of the healthy control group was 16.02±1.06. The mean age at menarche was 12.01±0.77 years. Thirty-one (81.6%) of the patients applied due to irregular menstrual cycle, five (13.2%) due to hirsutism, one (2.6%) due to acne, and one (2.6%) due to inguinal pain. The median menstrual cycle duration was 60 (15-200) days. Tanner staging of the patients: seven (18.4%) patients had stage IV, and 31 (81.5%) patients had stage V. According to mFGS, 30 (78.9%) of the patients had hirsutism (score >8), and 12 (31.5%) of the patients were obese. Table 1 summarizes the demographic and clinical characteristics of adolescents with PCOS. In the thyroid function tests of the patients, all of them were euthyroid, and 10 (26.2%) patients had dyslipidemia in the lipid profile. The mean homeostatic model assessment for insulin resistance (HOMA-IR) level was 3.42±1.53. The laboratory findings of PCOS patients are given in Table 2. Twenty-seven (71%) patients had polycystic ovary morphology (PCOM) on ultrasonography.

**Table 1:** The demographic and clinical characteristics of PCOS cases and the control group BMI: body mass index, DBP: diastolic blood pressure, mFGS: modified Ferriman Gallwey score, PCOS: polycystic ovary syndrome, SBP: systolic blood pressure, SDS: standard deviation score

Parameters	PCOS (n: 38)	Control Group (n: 40)	p values
Age (yrs)	15.99±1.18	16.02±1.06	0.273
Menarche age (yrs)	12.01±0.77	11.42±0.84	0.002
Menstrual cycle duration	60 (15-200)	30 (25-45)	<0.05
BMI SDS	0.81±1.69	0.16±0.73	0.029
SBP percentile	79.51±21.87	63.25±16.40	0.001
DBP percentile	77.85±21.86	56.15±17.53	<0.001
mFGS	10.5±3.07	3.25±1.64	<0.001
Waist:hip ratio	0.74±0.05	0.71±0.02	0.001

**Table 2:** The laboratory findings of PCOS cases DHEAS: dehydroepiandrosterone sulfate, E2: estradiol, FSH: follicle-stimulating hormone, LH: luteinizing hormone, PCOS: polycystic ovary syndrome

Parameters	PCOS (n: 38)
LH (IU/L)	17.56±11.80
FSH (IU/L)	6.05±1.50
E2 (pg/mL)	59.27±27.45
Total testosterone (ng/dL)	52.18+17.17
DHEAS (mg/dL)	348.01±124.54

The right-hand 2D:4D digit ratio was significantly lower (p<0.001) in the PCOS group (0.93±0.02) than in the control group (1.00±0.01). Similarly, the PCOS group had a significantly lower 2D:4D digit ratio in the left hand compared to the control group (0.98±0.03 vs. 1.00±0.01, p<0.001) (Table 3). In mFGS and finger ratios correlation analysis, no correlation was detected between the right-hand 2D:4D ratio and mFGS (r=-0.16, p=0.318). In contrast, a moderate negative correlation was detected between the left-hand 2D:4D ratio and mFGS (r=-0.53 p=0.01). No relationship was found between the patient's age at menarche, HOMA-IR levels, and digit ratios (p>0.05).

**Table 3:** Digit ratios of PCOS cases and the control group PCOS: polycystic ovary syndrome, 2D:4D: the ratio of the index finger to the ring finger

	PCOS (n: 38)	Control Group (n: 40)	p values
Left-hand 2D:4D	0.98±0.03	1.00±0.01	<0.001
Right-hand 2D:4D	0.93±0.02	1.00±0.01	<0.001

## Discussion

In this study, we explored the connection between anatomical markers of prenatal androgen exposure and PCOS. Our results demonstrated that PCOS patients had significantly lower both-hand 2D:4D ratios than healthy controls. This study was conducted based on the fact that PCOS represents prenatal androgen exposure. Hence, recognizing anatomic markers in adolescence might predict the later development of PCOS.

The difference between digit ratios and other sexual dysmorphism traits is that they are fixed in the prenatal period and do not change significantly in later periods. In humans, the relationship between second and fourth digit length is, on average, lower in males than in female adults [9], and thus digit ratio is considered sexual dimorphism [10]. This ratio is negatively associated with prenatal testosterone exposure, leading to a typically smaller ratio in males. Hönekopp et al.'s meta-analysis supports this relationship across children, adolescents, and adults [5]. Several studies have also revealed sex differences in digit ratio in fetuses and infants [11]. Studies conducted across various age groups of women have shown a reduced digit ratio in those with PCOS, suggesting this could be a useful early indicator [12-14]. In our study, we found that PCOS patients had significantly lower both-hand 2D:4D ratios than healthy controls. Although the prenatal history of our patients is not clearly known, this result suggests that there may be antenatal androgen exposure in PCOS patients.

A link was found in a study between a low digit ratio and delayed age of menarche, which implies higher androgen exposure during fetal development [15,16]. They suggested their findings supported the fetal origins hypothesis, highlighting the ability of reproductive organs to adapt and change during development due to the hormonal environment in utero. The ability of an organism to adapt and change its phenotype in response to environmental factors, while still adhering to its genetic potential, seems to lead to lasting alterations in the characteristics related to the onset of menstruation, an essential component of an organism's life cycle [16]. Our study did not find a case of delayed menarche, and no relationship was found between age at menarche and digit ratios.

A study showed a positive correlation between 2D:4D ratios and estradiol levels from single measurements taken throughout the menstrual cycle [17]. Additionally, 2D:4D ratios are found to be inversely related to fetal testosterone levels, as determined by amniocentesis [18]. Furthermore, it has been reported that women with excessive testosterone exposure before birth have a lower digit ratio (masculine) than women in the control group. On the other hand, 46 XY males who were completely insensitive to androgens had higher (feminine) ratios than control healthy males [19]. However, the evidence linking these ratios to adult circulating hormone levels is still uncertain. Women with the virilizing type of congenital adrenal hyperplasia (CAH) due to 21-hydroxylase deﬁciency are prenatally exposed to high androgens from the adrenal glands. Two studies observed that women with CAH have lower right-hand 2D:4D ratios compared to healthy women [20,21]. In our patient group, we found a negative correlation between hirsutism, a clinical finding of hyperandrogenism, and left-hand 2D:4D ratios.

Limitations

This study has some limitations, including its single-institution design and small sample size, which may not represent the general population. The cross-sectional nature of the study prevents determining cause-and-effect relationships. Additionally, laboratory data of the study were obtained retrospectively.

## Conclusions

Our study revealed a noteworthy decrease in the digit ratio among the PCOS group. This finding suggests an increase in androgen exposure and underscores the potential for early estimation, a key implication of our research. Hence, recognizing anatomic markers in adolescence might predict the later development of PCOS.
